# Effect of air pollution on online medical consultations for ocular surface diseases in China

**DOI:** 10.3389/fpubh.2025.1627435

**Published:** 2025-11-26

**Authors:** Chenyi Kang, Xiaomeng Yuan, Siqi Yan

**Affiliations:** 1 School of Public Policy and Administration, Xi’an Jiaotong University, Xi’an, China; 2 Department of Ophthalmology, The Second Affiliated Hospital of Xi’an Jiaotong University, Xi’an, China; 3 Institute for Stem Cell & Regenerative Medicine, The Second Affiliated Hospital of Xi’an Jiaotong University, Xi’an, China; 4 Key Laboratory of Environment and Genes Related to Diseases of Ministry of Education of China, Xi’an Jiaotong University, Xi’an, China

**Keywords:** air pollution, ocular surface diseases, online health consultations, spatiotemporal analysis, China

## Abstract

**Background:**

Ocular Surface Diseases (OSDs), such as dry eye disease and conjunctivitis, pose significant public health challenges globally, especially in China where they affect millions and lead to substantial economic costs. With the development of digital technology, online consultations have become increasingly popular, yet the association between environmental factors and the volume of online consultations for OSDs remains unclear. This study explores the impact of environmental factors, particularly air pollution, on the volume of online consultations for OSDs in China.

**Methods:**

We used web crawler technology to obtain OSDs online consultation data from China’s leading online medical platform “Good Doctor Online” from 2015–2019, and used correlation methods and fixed-effects regression models to analyze the impact of air pollution on the volume of online consultations for OSDs, and its potential regional variations and seasonal trends.

**Results:**

The results reveal significant positive correlations between online consultations for OSDs and air pollutants, notably PM_10_, and NO_2_ (*p* < 0.05). Regression analyses demonstrate that Air Quality Index (AQI) and PM_10_ significantly affect online consultation volumes, indicating that worsening air quality directly alters health-seeking behavior. Additionally, the impact of air pollution on online consultations shows regional variability: AQI, PM_2.5_, and CO were notably impactful in the Fenwei Plain, SO_2_ in the Jiangsu-Zhejiang-Shanghai region, and NO₂ in the Beijing-Tianjin-Hebei and Zhujiang Delta regions. Seasonal trends also showed peaks in consultation volumes corresponding with periods of high pollution, particularly during summer and winter.

**Conclusion:**

Our findings demonstrate that air pollution is positively associated with online consultations for OSDs, with AQI and PM_10_ showing particularly robust effects. The impacts vary by region—different pollutants play a dominant role in different areas—and consultations tend to peak during highly polluted summer and winter seasons.

## 1 Introduction

Ocular Surface Diseases (OSDs), including dry eye disease (DED) and conjunctivitis, pose significant public health challenges both globally and within China (1–3). DED, for instance, affects millions worldwide, with a notably higher prevalence in Asia—estimated at around 20.1% (4). In China, the prevalence of DED reaches approximately 31.4%, affecting about 394 million individuals nationwide (5, 6). OSDs not only impair patients’ health and quality of life but also impose considerable economic burdens. In 2008, the average annual treatment cost for a dry eye patient in the United States was $11,302, culminating in a total societal cost of $55.4 billion (7). Similarly, in China, annual healthcare expenditures related to DED are estimated to range from $104.2 billion to $166.6 billion, placing substantial strain on the country’s healthcare system (6). Therefore, implementing effective measures to combat OSDs is crucial not only for enhancing population health but also for contributing to sustainable development goals (8).

Air pollution has been identified as a major contributor to OSDs, affecting the ocular surface through mechanisms such as increased inflammation, oxidative stress, and tear film instability (9–13, 38). However, the etiology of OSDs is complex and influenced by a multitude of environmental risk factors, many of which remain poorly understood and warrant further investigation.

In terms of healthcare service provision, the development and adoption of online consultation technologies and platforms have been pivotal in enhancing the accessibility of medical services (14–16). Online consultation platforms offer significant advantages, including convenience, reduced exposure to environmental pollutants, and immediate access to specialist advice (17–19). For patients with OSDs, online consultations can be particularly beneficial. OSDs often require ongoing management and frequent consultations to adjust treatment plans (20, 21). Online platforms allow patients to communicate with healthcare providers without the need to expose themselves to environmental pollutants that could exacerbate their conditions (22, 23). This is especially relevant in areas with high levels of air pollution, where outdoor exposure may worsen ocular symptoms.

While the impact of air pollution on the incidence and severity of OSDs has been well-documented, indicating that poor air quality can lead to increased medical consultations (24, 25), the influence of other environmental factors on the demand for online consultations remains less clear. These factors may overlap with or differ from the risk factors for the diseases themselves and can affect the demand for online consultations directly by influencing patient behavior or indirectly by affecting the incidence rate of OSDs. For example, during periods of high air pollution, patients might prefer online consultations to avoid exposure to harmful outdoor air. Concurrently, the incidence of OSDs might rise due to the adverse effects of pollution, leading to a simultaneous increase in online consultation volume (26, 27).

**Figure 1:**
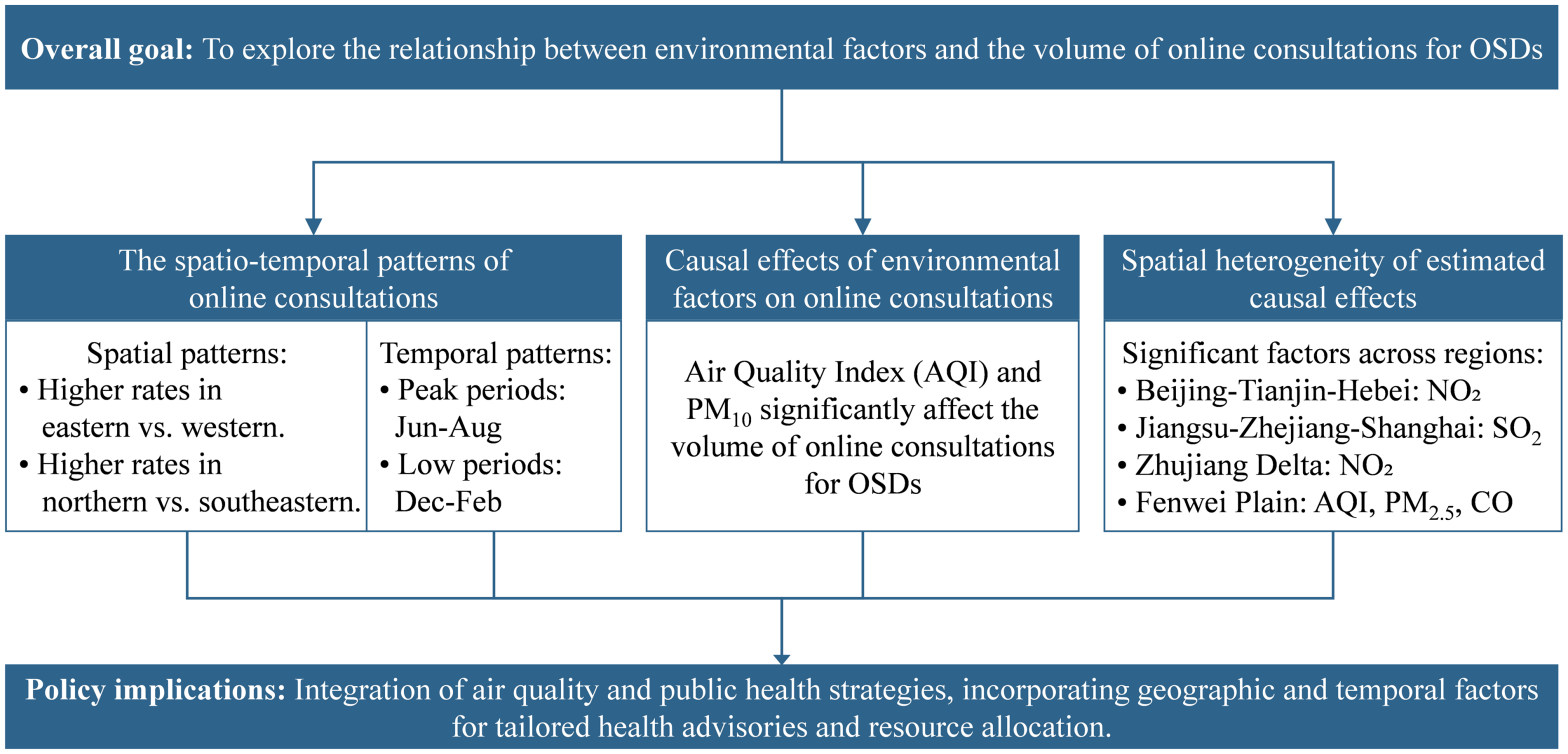
Research framework.

Given the complexity of these interactions, this study aims to explore the relationship between environmental factors—particularly air pollution—and the volume of online consultations for OSDs. Figure 1 illustrates the research framework of this paper. By analyzing detailed consultation data from the “Good Doctor Online,” a leading Chinese online consultation platform, this study first elucidates the spatial and temporal patterns of online consultations for OSDs. Then, we estimate the causal effects of various environmental factors on online consultations for OSDs using rigorous statistical methods. Furthermore, we analyze the spatial heterogeneity of these estimated effects to provide a more nuanced understanding of regional variations.

The contributions of this study are multifaceted. First, it provides a comprehensive empirical analysis of the impact of environmental factors on the demand for online medical consultations for OSDs, an area that has received limited attention in the literature. Second, by focusing on China—a country with significant variations in air pollution levels and a rapidly growing online healthcare market—the study offers valuable insights that are globally relevant, especially for countries experiencing similar environmental challenges. Third, the study adds to ongoing discussions on how environmental risks influence healthcare demand in the digital age, thereby contributing to both the environmental health and telemedicine literature.

## 2 Methods

### 2.1 Data

Our study utilizes three primary types of data: online consultations for OSDs, air quality indicators, and meteorological factors. This comprehensive dataset enables us to explore the relationships between environmental factors, particularly air pollution, and the volume of online consultations for OSDs.

The primary data source for online consultation metrics is the “Good Doctor Online” platform,^1^ a leading entity in internet healthcare in China. This platform integrates services such as offline and home-based consultations, online prescription services, and home medication delivery. By January 2024, it included over 10,000 hospitals and 820,000 physicians, facilitating significant patient-physician interactions crucial for our analysis.

We developed a web crawler on February 8, 2024, to collect online consultation data from the platform. To obtain comprehensive data, we crawled all ophthalmologists’ online consultation records, including patient demographics, consultation timings, and reported symptoms. Supplementary Figure A1 illustrates examples of the extracted data, depicting different symptoms at various times and locations.

Initially, we collected a total of 263,524 consultation records. We then performed data cleaning and preprocessing steps: (1) Data cleaning: excluded 7,672 records with missing key information. (2) Keyword filtering: the keyword list was developed based on typical OSDs-primarily DED and conjunctivitis-as well as their frequently associated symptoms (see the note of Supplementary Figure A1). We filtered the data by selecting records in which the “symptoms reported” label contained at least one of these predefined keywords, yielding a total of 29,883 valid records. (3) Timeframe selection: extracted consultation data spanning from 2015 to 2019 to exclude the disruptive impact of Coronavirus disease 2019 (COVID-19) on online consultations, thereby preventing bias in our analysis. After these processing steps, the final sample comprised 16,380 consultation records. We aggregated these records to obtain monthly and annual counts of online consultations for OSDs in prefecture-level cities for subsequent analysis.

We obtained daily Air Quality Index (AQI) values and concentrations of atmospheric pollutants—including PM_2.5_, PM_10_, SO_2_, NO_2_, and CO—for prefecture-level cities across China. These data are officially released by the China National Environmental Monitoring Center (CNEMC) and integrated into the China Stock Market & Accounting Research (CSMAR) Database. The air quality data spans the same period as the consultation data and was aggregated into monthly and annual statistics to align with our analysis periods. As the air quality data are reported at the prefecture level, we matched them with our OSD online consultations data using prefecture administrative codes, ensuring spatial consistency^2^.

Meteorological data—including precipitation, temperature, humidity, sunshine duration, wind speed, and wind direction—were sourced from the National Oceanic and Atmospheric Administration (NOAA). Similar to the air quality data, these variables were processed into monthly and annual aggregates for consistency. The specific meteorological variables are defined as follows: (1) Precipitation: Monthly accumulated precipitation in the city (millimeters). (2) Temperature: Includes average monthly temperature, monthly minimum temperature, and monthly maximum temperature (degrees Celsius). (3) Relative Humidity: Average monthly relative humidity in the city (%). (4) Sunshine Duration: Total monthly sunshine duration in the city (hours). (5) Wind Speed: Average monthly wind speed (meters per second). (6) Wind Direction: Includes maximum wind direction, ratio of maximum wind direction, extreme wind direction, and ratio of extreme wind direction. Wind directions are categorized into four primary groups for analysis.

### 2.2 Statistical methods

The statistical methods applied include:

(1) Descriptive statistics: descriptive statistics were used to summarize the overall characteristics of the data. Supplementary Table A2 provides an overview of the data distribution, including means, standard deviations, and the number of observations for each variable.
(2) Pearson correlation analysis: we conducted Pearson correlation analysis to qualitatively assess the associations between the online consultation rates for OSDs and environmental factors, particularly air pollution indices. This analysis was performed at both the national level and within key regions, including: Beijing-Tianjin-Hebei region, the Jiangsu-Zhejiang-Shanghai region, the Zhujiang Delta region (also called the Pearl River Delta Region), and the Fenwei Plain region. The cities included in each of the four regions are listed in the Supplementary Table A1.

These regions are focal points in China’s national air pollution control action plans issued by the State Council, including the “Action Plan for Air Pollution Prevention and Control” (2013), the “Three-Year Action Plan to Fight Air Pollution” (2018), and the “Action Plan for Continuous Improvement of Air Quality” (2023).

The correlation analysis helps in identifying preliminary relationships between variables, which is essential before conducting regression analysis.

(3) Regression analysis. To quantitatively estimate the causal impact of air pollution on the volume of online consultations for OSDs, we employed panel data regression analysis using a fixed-effects model. Specifically, we have constructed Equation 1, and the model is as follows:

(1) $$OC_{\mathrm{it}}=\alpha +\beta_{1}\text{Pollutio}n_{\mathrm{it}}+\beta_{2}\text{Control}s_{\mathrm{it}}+\mu_{i}+\lambda_{\mathrm{pq}}+\varepsilon_{\mathrm{it}}$$

where subscripts i , t , p and q denote city, month, province and quarter, respectively. The dependent variable $OC_{\mathrm{it}}$ is the rate of online consultations for OSDs of city i in month t , which is measured by the ratio of online consultations to the total number of corresponding urban Internet users. $\text{Pollutio}n_{\mathrm{it}}$ is the independent variable, which includes logged values of air quality indicators (e.g., AQI, PM_10_, PM_2.5_, SO_2_, NO_2_, and CO). $\text{Control}s_{\mathrm{it}}$ is a series of control variables, which encompasses some meteorological factors. Referring to Auffhammer and Kellogg (28), we incorporate variables such as temperature (maximum, minimum and average temperatures), humidity, sunshine duration, wind speed, and wind direction (maximum wind direction and extreme wind direction) into the control variables to mitigate the influence of meteorological factors on air pollutants. $\mu_{i}$ represents city fixed effects, controlling for factors at the city level that do not change over time, such as geographic and economic characteristics. $\lambda_{\mathrm{pq}}$ represents province-quarter fixed effects, controlling for factors that vary over time but are consistent across cities within the same province and quarter, like seasonal effects and policy changes. $\varepsilon_{\mathrm{it}}$ is the error term.

Supplementary Table A2 presents the definition of key variables and their summary statistics.

## 3 Results

### 3.1 Spatial patterns of online consultations for OSDs

**Figure 2:**
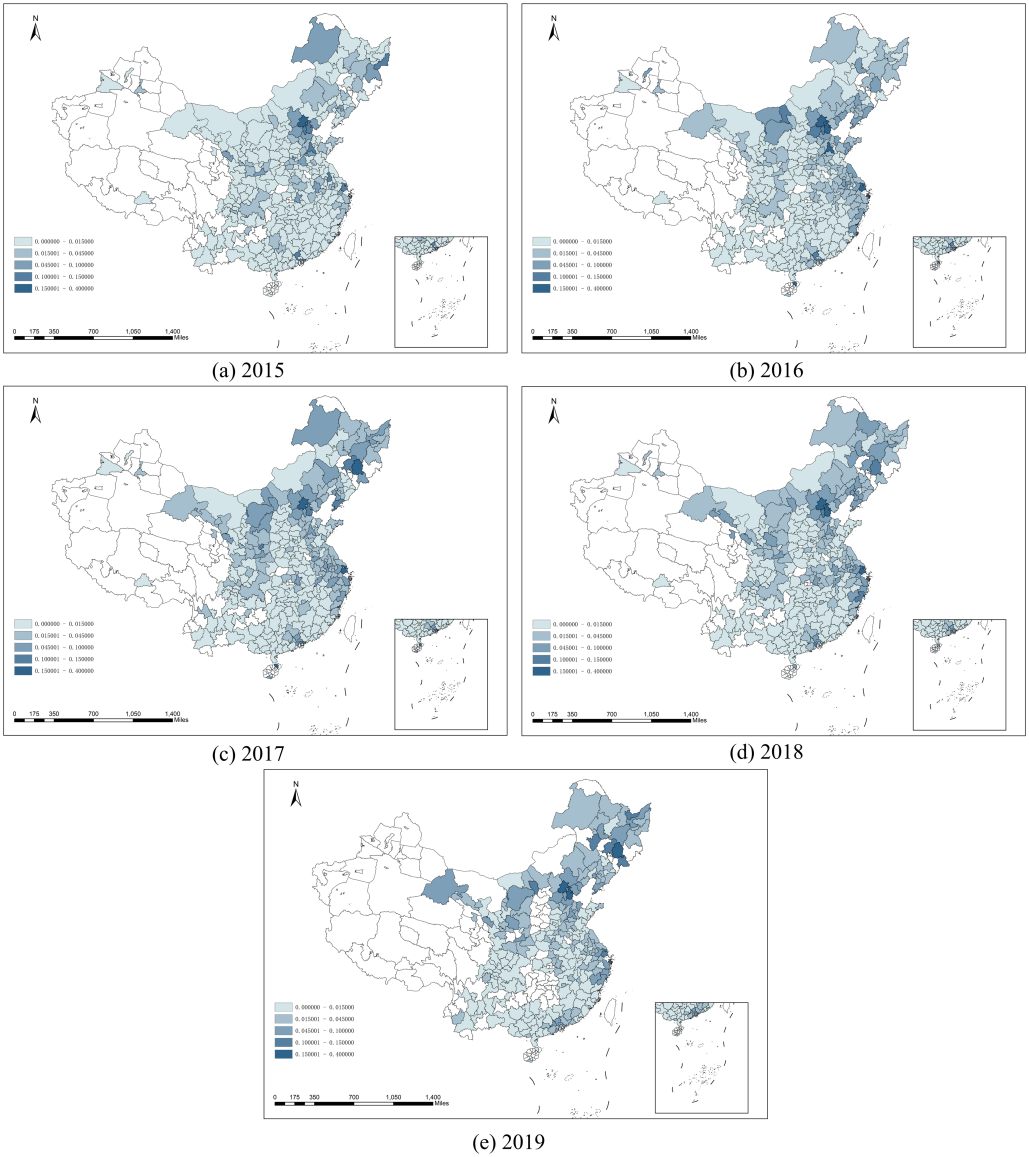
Spatial patterns of online consultations for OSDs in China from 2016 to 2019. **(a)** to **(e)** show the spatial distribution of online consultation for OSDs in China from 2015 to 2019.

Figure 2 illustrates the spatiotemporal distribution of online consultations for OSDs across prefecture-level cities in China. To mitigate the impact of population differences among these cities on the volume of online consultations, we have mapped the ratio of the annual OSDs online consultations to the total year-end population of each city. The darker the color, the higher the proportion of residents opting for online consultations in that region.

The spatial distribution of online consultations for OSDs, represented in Figure 2, shows marked disparities across China. The visualization, adjusted for population variances, indicates higher consultation rates in darker regions. Notably, eastern regions of China have higher rates compared to western regions, with particularly low rates in northwestern provinces like Xinjiang, Tibet, and Qinghai. This discrepancy can largely be attributed to differences in population density, internet infrastructure quality, and health awareness levels. In contrast, northern regions including North China and Northeast China display higher rates of online consultations than the southeastern coastal areas, possibly due to the harsher winter climates in the north that increase the prevalence of OSDs and make online consultations a more appealing option than in-person visits.

Figure 2 also depicts the change of spatial distribution over time from 2015 to 2019, showing significant growth in online consultation in the eastern coastal and northern regions. This rise is attributed to enhanced health awareness and improved internet infrastructure, facilitating greater access to online medical services. Despite this growth, the western regions continue to experience lower rates, underscoring an unmet need for medical resources in areas characterized by challenging geographical and climatic conditions.

Notably, there is a significant gap between the prevalence of OSDs and the online consultation rate in the western region, which may reveal the lack of medical resources in this area. The western region of China is characterized by high altitudes, cold and dry climates, and strong ultraviolet radiation, all of which are key factors influencing the prevalence of OSDs (29–33). Evidence suggests that the incidence of OSDs in the western region is higher than in other parts of China (34). However, the volume of online consultations for OSDs in the western region is significantly lower compared to other regions. This phenomenon indicates a mismatch between the high incidence of OSDs and the limited availability of online medical resources in the western region, leading to a lower demand for online consultations.

### 3.2 Seasonal variations of online consultations for OSDs

**Figure 3:**
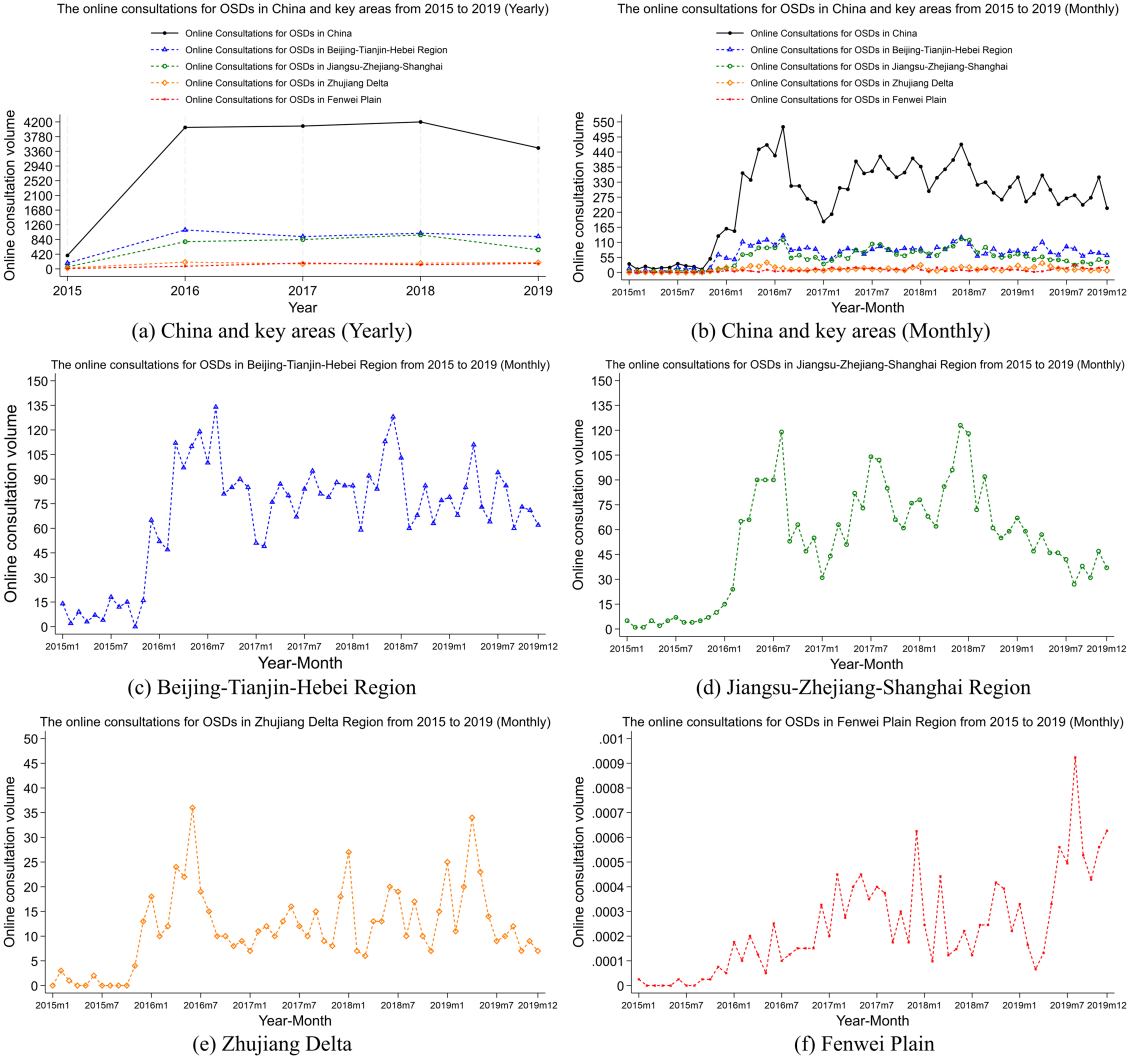
Trends in online consultation volume for OSDs. **(a)** to **(f)** show the yearly and monthly trends in online consultation volume for OSDs nationwide, as well as the monthly trends in the Beijing-Tianjin-Hebei region, Jiangsu-Zhejiang-Shanghai region, the Zhujiang Delta region and the Fenwei Plain region, respectively. The x-axis is the corresponding year-month, the y-axis is the number of online consultations for OSDs (persons).

**Figure 4:**
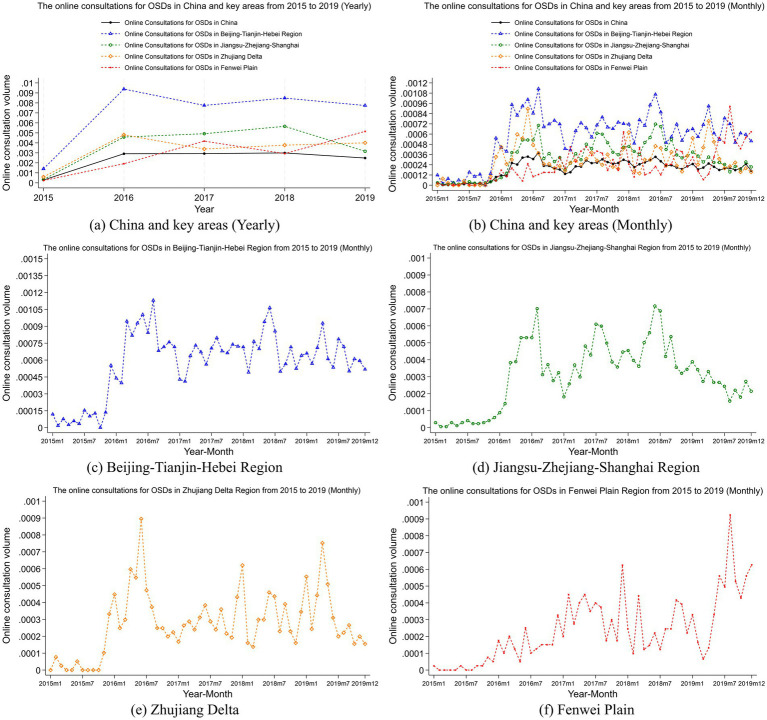
Trends in relative online consultation volume for OSDs. **(a)** to **(f)** show the yearly and monthly trends in relative online consultation volume for OSDs nationwide, as well as the monthly trends in the Beijing-Tianjin-Hebei region, Jiangsu-Zhejiang-Shanghai region, the Zhujiang Delta region and the Fenwei Plain region, respectively. The x-axis is the corresponding year-month, the y-axis is the rate of online consultations for OSDs. The rate of online consultations for OSDs is measured by the ratio of monthly consultations to the total population of the area.

Figures 3, 4 illustrate the annual and monthly trends in the number of online consultations and the proportion of online consultations for OSDs across China and four key regions, namely, the Beijing-Tianjin-Hebei region, Jiangsu-Zhejiang-Shanghai region, Zhujiang Delta region, Fenwei Plain region.

At the national level, the number of online consultations in China has stayed at a fairly stable level since 2016. Although our sample period starts in 2015, seasonal fluctuations in that year were relatively weak due to the overall low level of consultation rates. From 2016 onwards, the seasonal patterns became much clearer. In terms of the seasonal patterns, peak periods for online consultations often occur between June and August, possibly due to the high summer temperatures reducing people’s willingness to engage in outdoor activities, leading to an increase in online consultations. The low periods for online consultations occur between December and February. This trend may be attributed to a significant reduction in outdoor activities during winter, leading individuals to spend more time indoors. Consequently, this decreases in exposure to cold weather and dust storms may reduce the incidence of OSDs and, in turn, lower the demand for online consultations. Additionally, the Chinese New Year usually falls between late January and early February, representing the most important holiday of the year. During this festive period, people’s pace of life tends to slow down, resulting in decreased attention to health issues and, subsequently, a decline in consultation volume. The variation in the relative online consultation volume in Figure 4 aligns with the changes in absolute number in Figure 3, with the peak occurring annually from June to August, indicating significant seasonal fluctuations.

Given China’s vast geographical expanse and the significant differences in socioeconomic characteristics and air pollution levels across regions, we further analyzed four key regions: the Beijing-Tianjin-Hebei region, the Jiangsu-Zhejiang-Shanghai region, the Zhujiang Delta region, and the Fenwei Plain region. Figures 3b, 4b present the monthly online consultation volumes and rates in these key areas. The Beijing-Tianjin-Hebei region shows the highest monthly online consultation volumes, followed by the Jiangsu-Zhejiang-Shanghai region, while the Zhujiang Delta and Fenwei Plain regions exhibit relatively lower levels. This may be due to the higher population density, better internet infrastructure, and stronger health awareness in the Beijing-Tianjin-Hebei and Jiangsu-Zhejiang-Shanghai regions, leading to greater acceptance and accessibility of online consultation services. The humid climate in the Zhujiang Delta may result in a lower incidence of ocular diseases, which could influence the region’s lower online consultation levels. In contrast, the relatively underdeveloped internet infrastructure in the Fenwei Plain may impact the accessibility of online healthcare services, resulting in fewer online consultations in that region. Furthermore, Figure 4b reveals that the online consultation rates in the Beijing-Tianjin-Hebei, Jiangsu-Zhejiang-Shanghai, and Zhujiang Delta regions are all above the national average. The Fenwei Plain region initially had a rate below the national average but showed a gradual increase after 2019, eventually surpassing the national average. This suggests that the government should consider continuing to promote online healthcare models in this region, enhancing residents’ understanding and utilization of online medical services.

Figures 3c, 4c depict the trends in monthly online consultation volumes and rates in the Beijing-Tianjin-Hebei region. Similar to the national trend, the peak in online consultations occurs between June and August and the low periods is from December to February, with August 2016 marking the highest consultation volume in this region, after which the volume stabilizes. Figures 3d, 4d show the trends for the Jiangsu-Zhejiang-Shanghai region, where the peak online consultation period also falls between June and August and the low periods is from December to February, with the highest monthly consultation volume occurring in June 2018, followed by a decline. Figures 3e, 4e illustrate the trends for the Zhujiang Delta region, where the peak period is likewise from June to August and the low periods is from December to February, with the highest monthly consultation volume in June 2016. Figure 3f, 4f present the trends for the Fenwei Plain region, where the peak online consultation period is between December and February and the low periods is from June to August, with the highest monthly consultation volume recorded in August 2019. It is evident that, except for the Fenwei Plain, the other three key regions experience their peak online consultation periods in the summer, while the low periods are observed in the winter. In contrast, the Fenwei Plain experiences its peak in the winter and a trough in the summer, which may be attributed to differences in climatic conditions and pollution characteristics across regions.

### 3.3 Effects of influencing factors

#### 3.3.1 Associational relationship between air pollution and online consultations for OSDs

**Figure 5:**
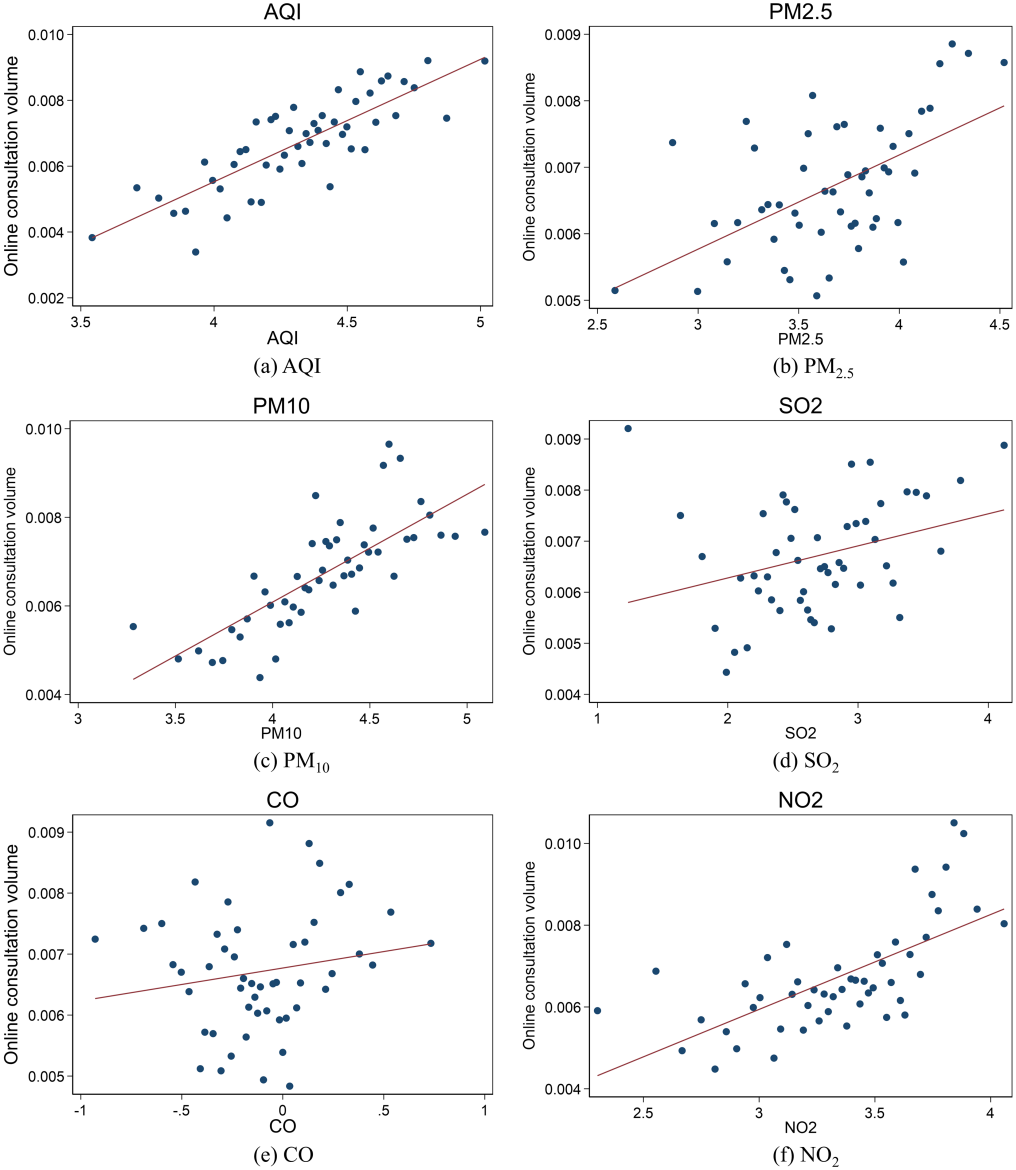
Scatter plot of air pollution and online consultations for OSDs. **(a)** to **(f)** show scatter plots of AQI, PM_2.5_, PM_10_, SO_2_, CO, NO_2_, and online consultations for OSDs, respectively. The x-axis represents the corresponding pollutant level, and the y-axis represents the number of online consultations for OSDs. A regression line is drawn in each subgraph to show the relationship between pollutants and the number of consultations.

Figure 5 shows the scatter plot between the number of online consultations for OSDs and AQI as well as five pollutants including PM_2.5_, PM_10_, SO_2_, CO, and NO_2_. AQI and air pollutants, especially PM_2.5_, PM_10_ and NO_2_, showed positive correlations with online consultations, indicating a negative impact of air pollution on OSD consultation.

Pearson’s correlation coefficient analysis was performed to evaluate the relationship between OSDs and independent variables. Columns (1)–(5) of Table 1 present the correlation analysis between the online consultation rates for OSDs and air pollution indices at both the national level and in four key regions, respectively. The results indicates that significant correlations are observed among most of these factors. At the national level, the online consultation rate for OSDs shows a significant positive correlation with AQI, PM_10_, and NO_2_ (*p* < 0.05), with correlation coefficients of 0.05, 0.02, and 0.04, respectively. In the Beijing-Tianjin-Hebei region, the online consultation rate shows negative correlations with PM_2.5_, PM_10_, SO_2_, and CO (*p* < 0.05). In the Jiangsu-Zhejiang-Shanghai region, the correlations between online consultation rate for OSDs and air pollution indicators are consistently negative, with all correlations being significant (*p* < 0.01). In the Zhujiang Delta, the online consultation rate is significantly positively correlated with NO_2_ (*p* < 0.01), with correlation coefficients of 0.17. In the Fenwei Plain, the online consultation rates for OSDs is significantly positively correlated with AQI and NO_2_ (*p* < 0.05), while SO_2_ has a negative correlation (*p* < 0.05).

**Table 1:** Correlation analysis: nationwide and key regions.

	(1)	(2)	(3)	(4)	(5)
	China	Beijing-Tianjin-Hebei region	Jiangsu-Zhejiang-Shanghai region	Zhujiang delta region	Fenwei plain region
Variable	*OC*	*OC*	*OC*	*OC*	*OC*
*OC*	1	1	1	1	1
*AQI*	0.05***	−0.05	−0.16***	0.05	0.08
*PM_2.5_*	−0.01	−0.08	−0.18***	−0.02	0.00
*PM_10_*	0.02	−0.20***	−0.17***	−0.03	0.02
*SO_2_*	−0.02	−0.39***	−0.13***	−0.05	−0.09
*CO*	−0.02	−0.16***	−0.16***	0.03	−0.05
*NO_2_*	0.04***	−0.02	−0.10***	0.17***	0.10***

***p < 0.01, **p < 0.05, *p < 0.1.

#### 3.3.2 Causal relationship: average effects

Table 2 shows the regression results. AQI and PM_10_ had significant positive effects on OSDs. AQI reflects the level of air quality, while PM_10_ is one of the indicators of particulate pollution that directly stimulates the ocular surface. Particulate matter can deposit on the ocular surface, causing mechanical damage and irritation, which increases the likelihood of OSDs such as dry eyes, itching, and congestion. AQI, representing overall air pollution, reflects the cumulative effects of various pollutants on the ocular surface, exacerbating their adverse impacts. Although pollutants like PM_2.5_, SO_2_, CO, and NO_2_ also showed positive relationships in the regression analysis, their coefficients did not achieve statistical significance, suggesting their effects on OSDs may be minimal or inconsistent.

**Table 2:** National average effects.

	(1)	(2)	(3)	(4)	(5)	(6)
Variable	*OC*	*OC*	*OC*	*OC*	*OC*	*OC*
*AQI*	0.0013* (1.87)					
*PM_2.5_*		0.0008 (1.29)				
*PM_10_*			0.0013* (1.79)			
*SO_2_*				0.0005 (1.16)		
*CO*					0.0007 (1.04)	
*NO_2_*						0.0004 (0.58)
Constant	0.0058 (0.98)	0.0093* (1.86)	0.0065 (1.19)	0.0107** (2.18)	0.0125*** (2.78)	0.0113** (2.32)
Controls	Yes	Yes	Yes	Yes	Yes	Yes
Observations	14,599	14,599	14,599	14,599	14,599	14,599
Adjusted R-squared	0.2783	0.2782	0.2783	0.2781	0.2781	0.2781
City FEs	Yes	Yes	Yes	Yes	Yes	Yes
Province-Quarter FEs	Yes	Yes	Yes	Yes	Yes	Yes

Standard errors, reported in parentheses, are clustered at the city level. ***p < 0.01, **p < 0.05, *p < 0.1. Bold value represents that this element has a significant impact on online consultations for OSDs.

#### 3.3.3 Causal relationship: heterogeneous effects among regions

**Figure 6:**
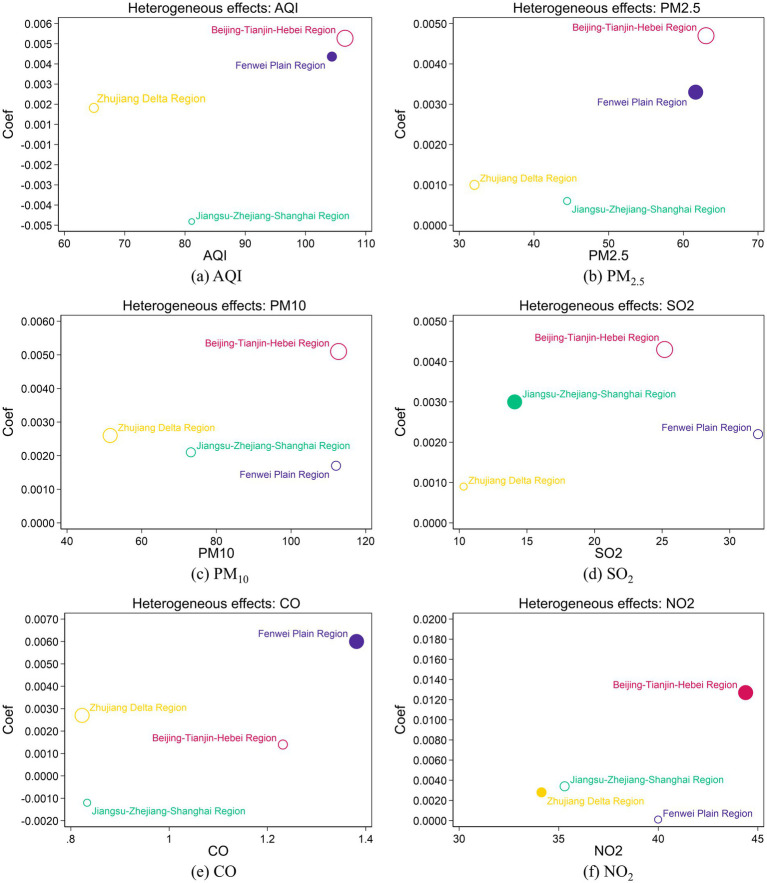
Heterogeneous effects. **(a)** to **(f)** show the heterogeneous effects of AQI, PM_2.5_, PM_10_, SO_2_, CO, NO_2_, respectively. The x-axis represents the average AQI and the average concentrations of pollutants (PM_2.5_, PM_10_, SO_2_, CO, NO_2_) for China and the four key regions during the sample period, while the y-axis represents the coefficients of the corresponding variables on the online consultation rate. The solid circle represents statistically significant coefficients, while hollow circle indicates non-significant ones. The size of the circle represents the magnitude of the coefficient.

Figure 6 illustrates the regional heterogeneous effects of AQI and five pollutants (PM_10_, PM_2.5_, SO_2_, CO, and NO_2_) on the online consultation for OSDs. The figure reveals substantial variation in the sensitivity to different pollutants across regions. The detailed regression results are presented in Supplementary Tables A3–A6.

Figure 6a shows the regional heterogeneity in the impact of AQI on online consultations for OSDs. It is evident that AQI significantly affects the online consultation volume in the Fenwei Plain, which is the region with high levels of AQI, indicating a link between air pollution severity and the demand for online consultations related to OSDs. Moreover, the average AQI level in the Beijing-Tianjin-Hebei region is similar to the Fenwei Plain. However, the impact of AQI on online consultations is not significant in the Beijing-Tianjin-Hebei region, which may be due to the following reasons. The coal combustion during the winter heating season in the Fenwei Plain results in more complex pollution components, leading to more severe health impacts. Additionally, the Fenwei Plain has relatively limited medical resources compared to the Beijing-Tianjin-Hebei region, prompting residents to rely more on online consultations. Consequently, despite similar AQI levels in these regions, the effects on online consultation for OSDs differ significantly.

Figure 6b illustrates the regional heterogeneity in the impact of PM_2.5_ on the online consultation for OSDs. It can be observed that the Fenwei Plain region, which experiences prolonged exposure to high concentrations of PM_2.5_, shows a significant effect of PM_2.5_ on the online consultation for OSDs. This may be attributed to the high PM_2.5_ levels resulting from wintertime coal combustion for heating and industrial pollution in the region. Long-term exposure to such elevated levels of PM_2.5_ poses a substantial risk to ocular health, thereby increasing the demand for online medical consultations among residents. In contrast, despite similarly high PM_2.5_ concentrations in the Beijing-Tianjin-Hebei region, the impact on online consultations for OSDs is not significant. This could be due to the availability of abundant offline medical resources and convenient transportation infrastructure in the area, which provide higher accessibility to in-person healthcare services, thus reducing the reliance on online consultations despite the high levels of PM_2.5_.

Figure 6c demonstrates the regional heterogeneity in the impact of PM_10_ on the online consultation for OSDs. It is evident that PM_10_ did not exert a significant influence across the four key regions. However, in the baseline regression, PM_10_ has been demonstrated to affect the national-level average online OSD consultation rate. This phenomenon may be attributable to the following reasons. On the one hand, the development level in the Jiangsu-Zhejiang-Shanghai region exceeds the national average, enabling residents to better access protective measures such as air purifiers, which may mitigate the impact of PM_10_ on eye health. On the other hand, the PM_10_ concentrations in the Beijing-Tianjin-Hebei region and the Fenwei Plain exceed the national average. This prolonged exposure to elevated PM_10_ levels among residents in these areas may lead to a degree of adaptation, thereby reducing the impact of pollutants on ocular health.

Figure 6d exhibits the regional heterogeneity in the impact of SO_2_ on the online consultation for OSDs. It is evident that SO_2_ has a significant effect on the online consultation for OSDs in the Jiangsu-Zhejiang-Shanghai region. This may be due to the high levels of industrial emissions in the region, leading to elevated SO_2_ concentrations. Given SO_2_’s strong irritant properties on the eyes, it can easily trigger ocular surface conditions, thereby increasing the demand for online consultations. Although the average SO_2_ concentrations in the Beijing-Tianjin-Hebei region, and the Fenwei Plain are higher than in the Jiangsu-Zhejiang-Shanghai region, SO_2_ does not have a significant impact on the online consultation for OSDs in these areas. This may be attributed to a potential non-linear relationship between SO_2_ concentration and health effects, where a significant impact on online consultation for OSDs may only occur when SO_2_ exposure reaches a specific threshold. Furthermore, differences in pollution sources and the extent to which residents have adapted to SO_2_ exposure across regions may also influence online consultation behaviors related to OSDs.

Figure 6e presents the regional heterogeneity in the impact of CO on the online consultation for OSDs. It indicates that CO has a significant effect on the online consultation for OSDs in the Fenwei Plain. This may be attributed to the high levels of CO pollution resulting from winter heating and industrial emissions in the region. Given CO’s strong irritant effects on the eyes and respiratory system, it likely increases the propensity for individuals with OSDs to seek online medical consultations.

Figure 6f depicts the regional heterogeneity in the impact of NO_2_ on the online consultation for OSDs. It reveals that NO_2_ has a significant effect on the online consultation for OSDs in the Beijing-Tianjin-Hebei and Zhujiang Delta regions. This may be because high concentrations of NO_2_ strongly irritate the ocular mucosa, leading to symptoms such as dry eyes, itching, and conjunctival congestion, thereby increasing the demand for online medical services for OSDs. Although the NO_2_ concentration in the Jiangsu-Zhejiang-Shanghai region is similar to that in the Zhujiang Delta, NO_2_ does not significantly affect the online consultation for OSDs in Jiangsu-Zhejiang-Shanghai region. This discrepancy may be due to differences in meteorological and geographical conditions between the two regions, leading to variations in pollutant exposure duration. The Zhujiang Delta experiences more pronounced temperature inversion events, which are unfavorable meteorological conditions that cause pollutants to accumulate near the ground, increasing the concentration and duration of exposure and thus amplifying NO_2_’s health effects. In contrast, in the Jiangsu-Zhejiang-Shanghai region, despite similar NO_2_ levels, better air circulation may prevent pollutants from lingering for extended periods, resulting in shorter actual exposure times and reducing NO_2_’s impact on OSDs. In the Fenwei Plain, the average NO_2_ concentration is higher than in the Zhujiang Delta but lower than in the Beijing-Tianjin-Hebei region. However, NO_2_ does not significantly affect the online consultation volume for OSDs in the Fenwei Plain. This may be because air pollution in the Fenwei Plain is not solely driven by NO_2_ but also includes other pollutants, such as PM_2.5_ and CO, which may overshadow or diminish the independent health effects of NO_2_.

## 4 Discussion

This comprehensive study has illuminated the multifaceted relationships between air pollution, meteorological conditions, and the prevalence of online consultations for OSDs in China. The findings reveal a significant interplay between environmental factors and health-seeking behaviors, particularly under adverse conditions, highlighting several key areas for public health intervention and policy development.

### 4.1 Impact of air pollution on health behavior

Extensive research has demonstrated that air pollution serves as a common risk factor for various chronic diseases, such as systemic lupus erythematosus, rheumatoid arthritis, ulcerative colitis and even endocrine disruption (35). Building upon this foundation, the data from this study supports the hypothesis that air pollution is a critical determinant in health-seeking behavior for individuals with OSDs. The presence of particulate matter (PM_2.5_ and PM_10_) and nitrogen dioxide (NO_2_) is robustly correlated with an increase in online consultations. This trend suggests that beyond being mere environmental nuisances, these pollutants are substantial health hazards that compel individuals to seek remote healthcare solutions. This likely serves as a protective behavior to avoid exacerbation of symptoms due to direct exposure to pollutants.

The increased responsiveness to air quality deterioration—evident from the spike in online consultations during periods of poor air quality—may also indicate a growing public awareness of the health impacts of pollution. This behavior is particularly pronounced in urban areas where there is better access to digital health platforms, suggesting that online health services are becoming a crucial buffer in public health strategies. For patients with chronic diseases, in-person visits often entail substantial time and financial costs, an issue that is especially pronounced in remote areas. Online consultation platforms help overcome geographical barriers, enabling patients to access specialized medical advice, which both provides better support to populations sensitive to air pollution and enhances the allocation efficiency of medical resources (36). This is especially relevant for managing chronic diseases like OSDs, which are directly impacted by environmental factors. This is particularly urgent in China, where despite some progress, outdoor PM₂.₅ pollution remained responsible for approximately 1.9 million deaths in 2021 (37).

### 4.2 Regional and seasonal variations

Our analysis identified significant regional disparities in the utilization of online consultations for OSDs. More urbanized and economically developed regions, particularly in Eastern China, reported higher frequencies of online health service usage. This disparity is influenced by several factors, including superior internet infrastructure, greater public awareness of health and technology, and higher levels of pollution.

Seasonal variations were also pronounced, with a notable increase in online consultations during the summer and winter months. This pattern is likely attributable to a combination of factors: high temperatures that impede travel, increased pollution from heating activities, atmospheric conditions that trap air pollutants close to the ground, and the inherent aggravation of OSDs symptoms during colder, drier conditions. These insights are critical for healthcare providers and policymakers, indicating a need for tailored health advisories and resource allocation that consider both geographic and temporal factors.

### 4.3 Implications for healthcare delivery and policy

The implications of these findings are significant for healthcare delivery systems and environmental health policies. There is a pressing need to integrate air quality improvement measures with broader public health strategies. Enhancing the infrastructure of telemedicine is also crucial, given the clear preference for online consultations during high pollution periods. Effective communication strategies are essential to inform at-risk populations about potential risks during high pollution periods, which can encourage preventive measures and timely medical consultations.

Policy-making must also adapt, with stricter air quality regulations and proactive pollution control measures needed to address the broader health implications of air quality. The indirect costs of pollution-related health service utilization should be factored into economic assessments of environmental regulations.

In conclusion, this study provides empirical evidence that air pollution significantly increases online consultations for OSDs, with notable regional heterogeneity (e.g., AQI, PM_2.5_ and CO in the Fenwei Plain, SO_2_ in the Jiangsu–Zhejiang–Shanghai region, and NO_2_ in the Beijing–Tianjin–Hebei region and Zhujiang Delta region) and seasonal peaks during summer and winter. These findings demonstrate how environmental factors shape health-seeking behavior in the digital age, thereby enriching both environmental health and telemedicine literature.

## 5 Conclusion

The impacts of air pollution on OSDs have been extensively studied. However, with the rapid development of digital healthcare, online consultations become an increasingly important channel for patients seeking medical services. The question of whether air pollution influences the online consultation behavior of patients with OSDs has yet to be fully explored. Investigating the association between air pollution and online consultations for OSDs not only helps to uncover the broader effects of air pollution on public health, but also provides valuable data for policymakers and digital healthcare platforms. Moreover, as air pollution worsens globally, understanding its potential impact on the demand for digital health services can assist in optimizing resource allocation, improving the accessibility and efficiency of healthcare services, and further advancing the growth of telemedicine.

This study has provided robust evidence that air pollution significantly influences health-seeking behavior, particularly for patients with OSDs. The shift towards online consultations in response to pollution exposure underscores the need for integrated approaches that combine environmental management with health infrastructure development. As such, understanding and addressing the environmental determinants of health behavior are critical for advancing public health outcomes in the face of ongoing environmental and climatic challenges.

The research highlights the necessity of viewing public health through an environmental lens, particularly for chronic diseases like OSDs. By embracing an integrated approach that combines environmental management, healthcare infrastructure development, and targeted public health interventions, policymakers can significantly improve health outcomes in contexts of increasing environmental challenges. The adaptation towards online healthcare, driven by environmental factors, not only reflects adaptability in healthcare delivery but also underscores an area ripe for policy innovation, aimed at better serving populations burdened by pollution and chronic health conditions. This study contributes to a deeper understanding of how environmental risks like air pollution shape healthcare behaviors and underscores the importance of integrating environmental health into broader public health and policy frameworks.

Further research is required to dissect the causal mechanisms linking specific pollutants to chronic diseases. Priority should be given to investigating whether complex mixtures of gaseous agents—such as nitrogen dioxide (NO_2_) and particulate matter of varying sizes (PM_2.5_, PM_10_, urban PM)—cumulatively worsen OSDs, as well as their potential effects on other chronic diseases, including systemic lupus erythematosus, rheumatoid arthritis, ulcerative colitis, and endocrine disorders. Longitudinal studies could deepen our understanding of the chronic impacts of exposure to various pollutants and refine the thresholds for public health advisories. In addition, it is essential to examine how exposure under different real-world conditions—including normal daily living, occupational settings, and environmental disasters—influences the development and severity of OSDs. Exploring the role of other environmental and climatic factors in influencing online health-seeking behaviors would also enrich our understanding of health behaviors in different environmental contexts.

## Data Availability

The data analyzed in this study is subject to the following licenses/restrictions: Unconstrained. Requests to access these datasets should be directed to CK, kangcheny@foxmail.com.

## Footnotes

1
www.haodf.com

2 According to the “Technical Regulation for Ambient Air Quality Assessment” issued by CNEMC, prefecture-level city air quality assessments are based on urban monitoring point data from the national ambient air quality monitoring network. Daily values represent 24-h averages across all monitoring sites within a city, while monthly values are calculated as the mean of daily averages. The CNEMC had established 1,436 state-controlled monitoring stations across 338 prefectural-level and higher cities by 2025, covering most urban areas and ensuring the representativeness of the monitoring data. In this study, we matched the air quality data with OSD online consultations data by prefecture administrative codes to ensure consistency in spatial scale.
