# Supplementary material for: Effect of air pollution on online medical consultations for ocular surface diseases in China
Source: Front Public Health. 2025 Nov 26;13:1627435. doi: 10.3389/fpubh.2025.1627435 (PMC12689930; doi:10.3389/fpubh.2025.1627435)
Supplement: Supplementary file 1 [file Data_Sheet_1.docx]

# Supplementary material

1. Examples of Extracted Consultation Data


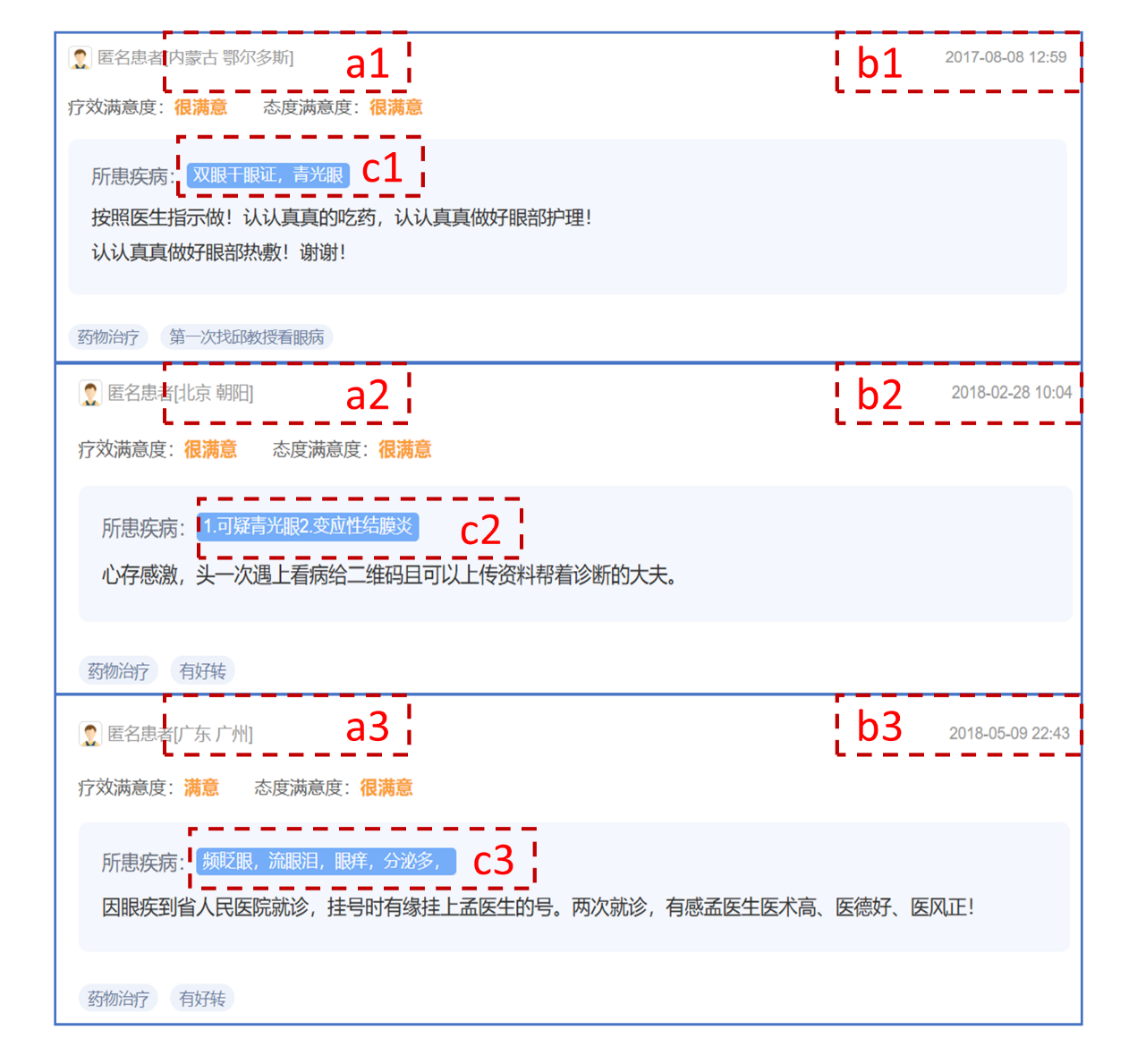


**Fig. A1** **Examples of Extracted Consultation Data**

*Notes*: The text in the figure is in Chinese. In the red dashed boxes, *a* indicates patient demographics, *b* indicates consultation timings, and *c* indicates symptoms reported. A record was retained if c (i.e. the "symptoms reported" label) contained at least one predefined keyword related to ocular surface diseases (OSDs). The examples depict the following symptoms in c: dry eye syndrome and glaucoma (c1); glaucoma and conjunctivitis (c2); and frequent blinking, excessive tearing, itchy eyes, and increased secretion (c3). All three examples were retained according to the keyword filter. The detailed keyword list includes symptoms such as dry eye syndrome, dry eye, conjunctivitis, conjunctiva, infectious conjunctivitis, corneal abrasion, corneal ulcer, keratitis, glaucoma, endophthalmitis, ophthalmic disease, itchy, allergic, acute, tearing, blinking infection, inflammation, congestion, redness, swelling, xerosis, epiphora, dacryocystitis, eye pain, irritation, photophobia, lacrimation, increased secretion, foreign body sensation, eye rubbing, surface eye mass, pterygium, pinguecula, etc.

2. Delineation of key areas

We select Beijing-Tianjin-Hebei region, Jiangsu-Zhejiang-Shanghai region, Zhujiang Delta region, and Fenwei Plain region as the key regions. Table A1 shows the prefecture-level cities included in each region.

**Table A1** Delineation of key areas.

| Area | City | Quantity |
| --- | --- | --- |
| Beijing-Tianjin-Hebei region | Beijing, Tianjin, Shijiazhuang, Tangshan, Qinhuangdao, Handan, Xingtai, Baoding, Zhangjiakou, Chengde, Cangzhou, Langfang, and Hengshui | 13 |
| Jiangsu-Zhejiang-Shanghai region | Nanjing, Wuxi, Xuzhou, Changzhou, Suzhou, Nantong, Lianyungang, Huai'an, Yancheng, Yangzhou, Zhenjiang, Taizhou, Suqian, Shanghai, Hangzhou, Ningbo, Wenzhou, Jiaxing, Huzhou, Shaoxing, Jinhua, Quzhou, Zhoushan, Taizhou, and Lishui | 25 |
| Zhujiang Delta region | Guangzhou, Foshan, Zhaoqing, Shenzhen, Dongguan, Huizhou, Zhuhai, Zhongshan, and Jiangmen | 9 |
| Fenwei Plain region | Xi'an, Xianyang, Weinan, Baoji, Tongch uan, Jinzhong, Lüliang, Yuncheng, Linfen, Luoyang, and Sanmenxia | 11 |

3. Definitions and descriptive statistics of main variables

**Table A2** Definitions and descriptive statistics of main variables.

| Variables | Definition | Mean | S.D. | Obs. |
| --- | --- | --- | --- | --- |
| **Dependent variable** |  |  |  |  |
| *OC* | The ratio of online consultations for OSDs to urban population | 0.0067 | 0.0163 | 16,380 |
| **Independent variables** |  |  |  |  |
| *AQI* | Log of monthly average AQI of the city | 4.3121 | 0.3432 | 17,210 |
| *PM_2.5_* | Log of monthly average PM_2.5_ concentrations of the city | 3.6551 | 0.5292 | 17,210 |
| *PM_10_* | Log of monthly average PM_10_ concentrations of the city | 4.2488 | 0.4749 | 17,210 |
| *SO_2_* | Log of monthly average SO_2_ concentrations of the city | 2.6851 | 0.6774 | 17,210 |
| *CO* | Log of monthly average CO concentrations of the city | -0.1099 | 0.3916 | 17,210 |
| *NO_2_* | Log of monthly average NO_2_ concentrations of the city | 3.3198 | 0.4554 | 17,210 |
| **Control variables** |  |  |  |  |
| *Precipitation* | Monthly accumulated precipitation of the city | 89.3154 | 92.9293 | 17,160 |
| *Temperature* | Average monthly temperature of the city | 14.6997 | 10.4448 | 17,160 |
| *Humidity* | Average monthly humidity of the city | 69.6814 | 13.3481 | 17,160 |
| *Wind speed* | Average monthly wind speed of the city | 4.9293 | 1.1487 | 17,160 |
| *Minimum temperature* | Monthly minimum temperature of the city | 10.0080 | 11.0571 | 17,160 |
| *Maximum temperature* | Monthly maximum temperature of the city | 19.5466 | 10.1040 | 17,160 |
| *Sunshine duration* | Monthly sunshine duration of the city | 164.5203 | 63.3145 | 17,160 |
| *Maximum wind direction 1 (N, NNE, NE, EN)* | =1 if the prevailing direction of the maximum wind speed corresponds to wind direction 1; = 0 otherwise | 0.3040 | 0.4600 | 15,287 |
| *Maximum wind direction 2 (E, ESE, SE, SSE)* | =1 if the prevailing direction of the maximum wind speed corresponds to wind direction 2; = 0 otherwise | 0.2441 | 0.4295 | 15,287 |
| *Maximum wind direction 3 (S, SSW, SW, WSW)* | =1 if the prevailing direction of the maximum wind speed corresponds to wind direction 3; = 0 otherwise | 0.2371 | 0.4253 | 15,287 |
| *Maximum wind direction 4 (W, WNW, NW, NNW)* | =1 if the prevailing direction of the maximum wind speed corresponds to wind direction 4; = 0 otherwise | 0.2148 | 0.4107 | 15,287 |
| *Extreme wind direction 1 (N, NNE, NE, EN)* | =1 if the prevailing direction of the extreme wind speed corresponds to wind direction 1; = 0 otherwise | 0.3271 | 0.4692 | 15,287 |
| *Extreme wind direction 2 (E, ESE, SE, SSE)* | =1 if the prevailing direction of the extreme wind speed corresponds to wind direction 2; = 0 otherwise | 0.2437 | 0.4293 | 15,287 |
| *Extreme wind direction 3 (S, SSW, SW, WSW)* | =1 if the prevailing direction of the extreme wind speed corresponds to wind direction 3; = 0 otherwise | 0.2352 | 0.4241 | 15,287 |
| *Extreme wind direction 4 (W, WNW, NW, NNW)* | =1 if the prevailing direction of the extreme wind speed corresponds to wind direction 4; = 0 otherwise | 0.1941 | 0.3955 | 15,287 |
| *Ratio of maximum wind direction 1 (N, NNE, NE, EN)* | The ratio of wind direction 1 to the direction of maximum wind speed | 0.2913 | 0.1835 | 15,287 |
| *Ratio of maximum wind direction 2 (E, ESE, SE, SSE)* | The ratio of wind direction 2 to the direction of maximum wind speed | 0.2366 | 0.1565 | 15,287 |
| *Ratio of maximum wind direction 3 (S, SSW, SW, WSW)* | The ratio of wind direction 3 to the direction of maximum wind speed | 0.2334 | 0.1579 | 15,287 |
| *Ratio of maximum wind direction 4 (W, WNW, NW, NNW)* | The ratio of wind direction 4 to the direction of maximum wind speed | 0.2387 | 0.1636 | 15,287 |
| *Ratio of extreme wind direction 1 (N, NNE, NE, EN)* | The ratio of wind direction 1 to the direction of extreme wind speed | 0.2986 | 0.1740 | 15,287 |
| *Ratio of extreme wind direction 2 (E, ESE, SE, SSE)* | The ratio of wind direction 2to the direction of extreme wind speed | 0.2359 | 0.1466 | 15,287 |
| *Ratio of extreme wind direction 3 (S, SSW, SW, WSW)* | The ratio of wind direction 3 to the direction of extreme wind speed | 0.2294 | 0.1518 | 15,287 |
| *Ratio of extreme wind direction 4 (W, WNW, NW, NNW)* | The ratio of wind direction 4 to the direction of extreme wind speed | 0.2362 | 0.1523 | 15,287 |

Notes: We consolidate the 16 wind directions into four primary categories and generate corresponding dummy variables.

4. Regression results for regional heterogeneity

**Table A3** Regional heterogeneous effects: Beijing-Tianjin-Hebei region

|  | (1) | (2) | (3) | (4) | (5) | (6) |
| --- | --- | --- | --- | --- | --- | --- |
| Variable | OC | OC | OC | OC | OC | OC |
| *AQI* | 0.0052 |  |  |  |  |  |
|  | (1.51) |  |  |  |  |  |
| *PM_2.5_* |  | 0.0047 |  |  |  |  |
|  |  | (1.24) |  |  |  |  |
| *PM_10_* |  |  | 0.0051 |  |  |  |
|  |  |  | (1.72) |  |  |  |
| *SO_2_* |  |  |  | 0.0043 |  |  |
|  |  |  |  | (1.02) |  |  |
| *CO* |  |  |  |  | 0.0014 |  |
|  |  |  |  |  | (0.41) |  |
| *NO_2_* |  |  |  |  |  | **0.0127**** |
|  |  |  |  |  |  | **(2.37)** |
| Constant | 0.0227 | 0.0324 | 0.0301 | 0.0322 | 0.0503 | 0.0105 |
|  | (0.54) | (0.87) | (0.93) | (0.77) | (1.64) | (0.29) |
| Controls | Yes | Yes | Yes | Yes | Yes | Yes |
| Observations | 696 | 696 | 696 | 696 | 696 | 696 |
| Adjusted R-squared | 0.6062 | 0.6061 | 0.6063 | 0.6062 | 0.6049 | 0.6095 |
| City FEs | Yes | Yes | Yes | Yes | Yes | Yes |
| Province-Quarter FEs | Yes | Yes | Yes | Yes | Yes | Yes |

Notes: Standard errors, reported in parentheses, are clustered at the city level. ***p <0.01, **p <0.05, *p <0.1.

**Table A4** Regional heterogeneous effects: Jiangsu-Zhejiang-Shanghai region

|  | (1) | (2) | (3) | (4) | (5) | (6) |
| --- | --- | --- | --- | --- | --- | --- |
| Variable | OC | OC | OC | OC | OC | OC |
| *AQI* | -0.0048 |  |  |  |  |  |
|  | (-1.54) |  |  |  |  |  |
| *PM_2.5_* |  | 0.0006 |  |  |  |  |
|  |  | (0.35) |  |  |  |  |
| *PM_10_* |  |  | 0.0021 |  |  |  |
|  |  |  | (1.12) |  |  |  |
| *SO_2_* |  |  |  | **0.0030***** |  |  |
|  |  |  |  | **(2.94)** |  |  |
| *CO* |  |  |  |  | -0.0012 |  |
|  |  |  |  |  | (-0.43) |  |
| *NO_2_* |  |  |  |  |  | 0.0034 |
|  |  |  |  |  |  | (1.46) |
| Constant | 0.0566** | 0.0231* | 0.0150 | 0.0155 | 0.0264** | 0.0134 |
|  | (2.28) | (1.78) | (0.93) | (1.66) | (2.65) | (1.07) |
| Controls | Yes | Yes | Yes | Yes | Yes | Yes |
| Observations | 1,392 | 1,392 | 1,392 | 1,392 | 1,392 | 1,392 |
| Adjusted R-squared | 0.4019 | 0.3994 | 0.4000 | 0.4022 | 0.3995 | 0.4015 |
| City FEs | Yes | Yes | Yes | Yes | Yes | Yes |
| Province-Quarter FEs | Yes | Yes | Yes | Yes | Yes | Yes |

Notes: Standard errors, reported in parentheses, are clustered at the city level. ***p <0.01, **p <0.05, *p <0.1.

**Table A5** Regional heterogeneous effects: Zhujiang Delta region

|  | (1) | (2) | (3) | (4) | (5) | (6) |
| --- | --- | --- | --- | --- | --- | --- |
| Variable | OC | OC | OC | OC | OC | OC |
| *AQI* | 0.0014 |  |  |  |  |  |
|  | (0.59) |  |  |  |  |  |
| *PM_2.5_* |  | 0.0010 |  |  |  |  |
|  |  | (0.53) |  |  |  |  |
| *PM_10_* |  |  | 0.0026 |  |  |  |
|  |  |  | (1.46) |  |  |  |
| *SO_2_* |  |  |  | 0.0009 |  |  |
|  |  |  |  | (1.02) |  |  |
| *CO* |  |  |  |  | 0.0027 |  |
|  |  |  |  |  | (0.59) |  |
| *NO_2_* |  |  |  |  |  | **0.0028**** |
|  |  |  |  |  |  | **(2.72)** |
| Constant | 0.0276 | 0.0286 | 0.0204 | 0.0315 | 0.0337 | 0.0239 |
|  | (1.36) | (1.68) | (1.13) | (1.48) | (1.69) | (1.32) |
| Controls | Yes | Yes | Yes | Yes | Yes | Yes |
| Observations | 444 | 444 | 444 | 444 | 444 | 444 |
| Adjusted R-squared | 0.2437 | 0.2432 | 0.2458 | 0.2433 | 0.2444 | 0.2495 |
| City FEs | Yes | Yes | Yes | Yes | Yes | Yes |
| Province-Quarter FEs | Yes | Yes | Yes | Yes | Yes | Yes |

Notes: Standard errors, reported in parentheses, are clustered at the city level. ***p <0.01, **p <0.05, *p <0.1.

**Table A6** Regional heterogeneous effects: Fenwei Plain region

|  | (1) | (2) | (3) | (4) | (5) | (6) |
| --- | --- | --- | --- | --- | --- | --- |
| Variable | OC | OC | OC | OC | OC | OC |
| *AQI* | **0.0042*** |  |  |  |  |  |
|  | **(1.90)** |  |  |  |  |  |
| *PM_2.5_* |  | **0.0033**** |  |  |  |  |
|  |  | **(2.24)** |  |  |  |  |
| *PM_10_* |  |  | 0.0017 |  |  |  |
|  |  |  | (1.11) |  |  |  |
| *SO_2_* |  |  |  | 0.0022 |  |  |
|  |  |  |  | (1.01) |  |  |
| *CO* |  |  |  |  | **0.0060***** |  |
|  |  |  |  |  | **(4.11)** |  |
| *NO_2_* |  |  |  |  |  | 0.0001 |
|  |  |  |  |  |  | (0.03) |
| Constant | -0.0131 | -0.0016 | 0.0058 | 0.0013 | 0.0056 | 0.0138 |
|  | (-0.69) | (-0.13) | (0.46) | (0.07) | (0.60) | (0.87) |
| Controls | Yes | Yes | Yes | Yes | Yes | Yes |
| Observations | 648 | 648 | 648 | 648 | 648 | 648 |
| Adjusted R-squared | 0.1525 | 0.1519 | 0.1498 | 0.1509 | 0.1555 | 0.1493 |
| City FEs | Yes | Yes | Yes | Yes | Yes | Yes |
| Province-Quarter FEs | Yes | Yes | Yes | Yes | Yes | Yes |

Notes: Standard errors, reported in parentheses, are clustered at the city level. ***p <0.01, **p <0.05, *p <0.1.
